# A protoplast generation and transformation method for soybean sudden death syndrome causal agents *Fusarium virguliforme* and *F. brasiliense*

**DOI:** 10.1186/s40694-019-0070-0

**Published:** 2019-05-15

**Authors:** Mitchell G. Roth, Martin I. Chilvers

**Affiliations:** 1 Department of Plant, Soil and Microbial Sciences, Michigan State University, 1066 Bogue St., East Lansing, 48824 MI USA; 2 Genetics Graduate Program, Michigan State University, 567 Wilson Rd., East Lansing, 48824 MI USA

**Keywords:** *Fusarium*, Soybean SDS, Protoplast, Transformation

## Abstract

**Background:**

Soybean production around the globe faces significant annual yield losses due to pests and diseases. One of the most significant causes of soybean yield loss annually in the U.S. is sudden death syndrome (SDS), caused by soil-borne fungi in the *Fusarium solani* species complex. Two of these species, *F. virguliforme* and *F. brasiliense*, have been discovered in the U.S. The genetic mechanisms that these pathogens employ to induce root rot and SDS are largely unknown. Previous methods describing *F. virguliforme* protoplast generation and transformation have been used to study gene function, but these methods lack important details and controls. In addition, no reports of protoplast generation and genetic transformation have been made for *F. brasiliense*.

**Results:**

We developed a new protocol for developing fungal protoplasts in these *Fusarium* species and test the protoplasts for the ability to take up foreign DNA. We show that wild-type strains of *F. virguliforme* and *F. brasiliense* are sensitive to the antibiotics hygromycin and nourseothricin, but strains transformed with resistance genes displayed resistance to these antibiotics. In addition, integration of fluorescent protein reporter genes demonstrates that the foreign DNA is expressed and results in a functional protein, providing fluorescence to both pathogens.

**Conclusions:**

This protocol provides significant details for reproducibly producing protoplasts and transforming *F. virguliforme* and *F. brasiliense*. The protocol can be used to develop high quality protoplasts for further investigations into genetic mechanisms of growth and pathogenicity of *F. virguliforme* and *F. brasiliense*. Fluorescent strains developed in this study can be used to investigate temporal colonization and potential host preferences of these species.

**Electronic supplementary material:**

The online version of this article (10.1186/s40694-019-0070-0) contains supplementary material, which is available to authorized users.

## Background

Soybean sudden death syndrome (SDS) is an economically important disease across soybean growing regions around the world [1–4]. Causal agents of soybean SDS are soil-borne ascomycete fungi in clade II of the *Fusarium solani* species complex [5, 6]. Two of the SDS-causing species, *F. virguliforme* and *F. brasiliense*, have been discovered in the U.S. [7, 8]. Although transformation of *F. virguliforme* has been demonstrated [9], it has not been for *F. brasiliense*. Due to the recent discovery of *F. brasiliense* on common bean (*Phaseolus vulgaris*) and soybean (*Glycine max*) roots in the U.S. [8, 10] and the significance of soybean SDS caused by these pathogens, it is desirable to have a detailed protocol for efficient genetic manipulation that is applicable to both pathogens. Improved understanding of the genetic underpinnings of SDS development could lead to improved management for these two pathogens.

Both *F. virguliforme* and *F. brasiliense* infect root tissues, with evidence suggesting that both appressoria and cell wall degrading enzymes are involved [11, 12]. However, the genetic mechanisms of SDS development are largely unknown. Two effector proteins have been identified and characterized from *F. virguliforme*, which seem to play a role in SDS development [13–15]. These two effector genes can also be found in isolates of *F. brasiliense* via BLAST searches, but the mechanism by which these effector proteins induce SDS remain unknown in both species. Breeding efforts have improved soybean tolerance to foliar symptom development, but no completely resistant lines exist to date [16]. Successful management of these pathogens can be achieved through long-term crop rotations and with seed treatments containing the fungicide fluopyram [17, 18], but long-term crop rotations are not widely adopted by growers, and different members of the *Fusarium solani* species complex have shown different sensitivities to fluopyram [19]. Therefore, improved genetic resistance is desired, but requires a deeper understanding of the genetic mechanisms of SDS development caused by each pathogen. Genome sequences are available for both of these pathogens [20, 21], but experimental identification and characterization of effector or other pathogenicity-associated genes through genetic manipulation needs to be done in order to gain a better understanding of how these pathogens induce soybean SDS.

One of the major challenges in genetic transformation of fungi is the development of high-quality protoplasts. Each fungus has unique cell wall properties, which can require different sets of expensive cell wall degrading enzymes to successfully remove the cell wall [22]. Once the cell wall is digested to reveal the membrane bound protoplast, it is prone to lysing or shriveling due to osmotic stress [22]. One protocol for protoplasting and transformation of *F. virguliforme* has been reported and used to generate a fluorescent strain [9], with additional studies using these methods to knock out candidate effector genes [14, 15] and genes involved in fungal development [23, 24]. No such protocols exist for *F. brasiliense*. Difficulties obtaining *F. brasiliense* and *F. virguliforme* protoplasts using the published protocol led us to investigate other methods. The objective of this study was to develop an efficient protoplasting and transformation method suitable for genetic manipulation in both *F. brasiliense* and *F. virguliforme* for future hypothesis testing of gene function.

## Methods

### Sensitivity to selectable antibiotics

**Fig. 1:**
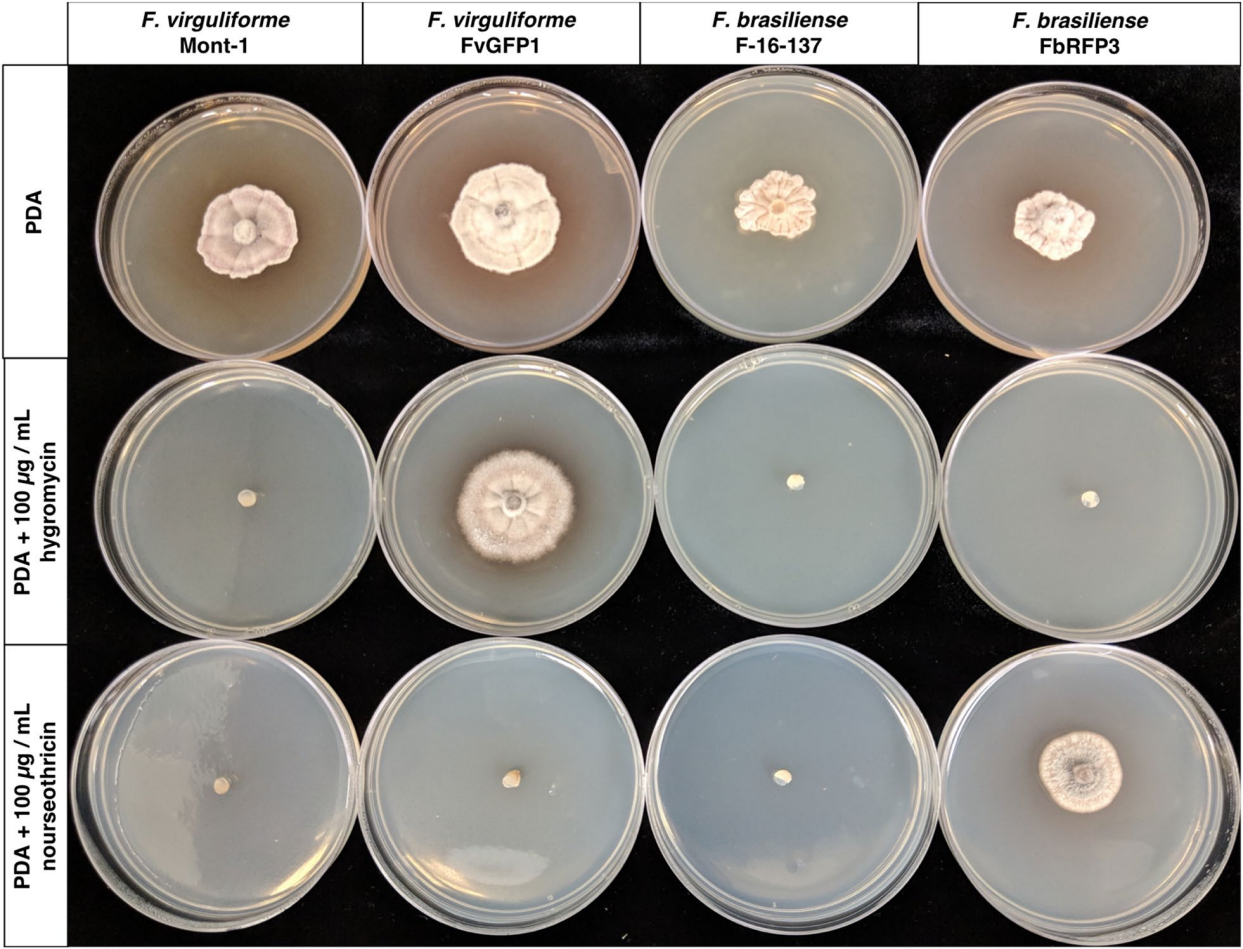
Wild type *F. virguliforme* and *F. brasiliense* strains are sensitive to hygromycin (100 µg/mL) and nourseothricin (100 µg/mL). Transgenic strains of *F. virguliforme* with the addition of the *hph* gene are resistant to hygromycin, while transgenic strains of *F. brasiliense* with the addition of the *nat* gene are resistant to nourseothricin

To determine an appropriate concentration to select for growth of successful transformants, the growth of wild type strains were tested in the presence of different concentrations of antifungal chemicals. The wild type strain of *F. virguliforme* chosen was Mont-1 (NRRL 22292), which was isolated from soybean in Illinois, and the wild type strain of *F. brasiliense* chosen was F-16-137, which was isolated from dry bean in Michigan. Full strength potato dextrose agar (PDA) (Neogen Corporation, Lansing, MI) was amended with 0, 25, 50, 100, or 150 µg/mL of hygromycin B (Millipore-Sigma, Burlington, MA) or nourseothricin (Research Products International, Mt. Prospect, IL). A single 0.5 mm PDA plug from 14-day old cultures of *F. virguliforme* isolate Mont-1 or *F. brasiliense* isolate F-16-137 were added to each amended plate. After 14 days, fungal growth was assessed visually to determine an appropriate concentration that provides 100% growth inhibition. Based on a visual assessment, 100 µg/mL of each antibiotic was sufficient to prevent fungal growth of both isolates (Fig. 1).

### Development of transformation constructs

DNA vectors for transformation were constructed to contain a resistance gene to a selectable antibiotic and a gene that provides a fluorescent phenotype when excited by specific wavelengths of light. Both transformation vectors were constructed with Gibson assembly [25] of four DNA fragments; a bacterial origin of replication, a bacterial selection marker, a fungal selection marker, and a fluorescent protein marker. A high copy origin of replication and a kanamycin resistance gene were cloned from a plasmid designed for the overexpression of MinC in cyanobacteria [26], which we refer to as pJM2016. For *F. virguliforme*, the selectable marker was the hygromycin phosphotransferase gene (*hph*) under the control of the *Aspergillus nidulans trpC* promoter and terminator derived from pCB1004 [27] and the fluorescent protein marker was enhanced green fluorescent protein (eGFP) under the control of the *A. nidulans gpd* promoter and *trpC* terminator, derived from pDS23-eGFP (M. Nowrousian, *unpublished*). For *F. brasiliense*, the selectable marker was the nourseothricin acetyltransferase gene (*nat*) under the control of the *A. nidulans trpC* promoter derived from pDS23-eGFP and the fluorescent protein marker was the red fluorescent protein mCherry, derived from pCMB-TMEr [28] under the control of the *A. nidulans gpd* promoter and *trpC* terminator. All primers used to amplify these regions prior to Gibson assembly were designed in this study and are provided in Additional file 1. Assembled plasmids were named Fv_GFP2 and pMCherry_NAT, respectively, and propagated in *Escherichia coli* strain DH5α plated on LB amended with 50 µg/mL kanamycin. Plasmids were isolated using the GeneJET Plasmid Miniprep kit (Thermo Fisher Scientific, Waltham, MA) and linearized with either SacI-HF or EcoRI-HF (New England Biolabs, Ipswich, MA) prior to transformation, according to the manufacturer protocol. The plasmid pmCherry_NAT is designed such that the selectable marker can be replaced by restriction digest with EcoRI and NheI, and the fluorescent marker gene can be replaced by restriction digest with NheI and SacI. A double digest of these plasmids can separate the transformation construct from the bacterial DNA sequences, which can be purified via gel extraction. Plasmid maps and sequences are available in Additional files 2 and 3.

### Protoplasting

A hybrid method of two existing protocols was used for developing protoplasts of *F. virguliforme* and *F. brasiliense* [9, 29]. A two to three-week-old PDA plate containing mature *Fusarium* sporodochia was flooded with 5 mL sterile water and scraped gently with a sterile spreader to dislodge spores. Approximately 4 mL of the water could be recovered from the plate and was filtered through two layers of sterile cheesecloth into a 15 mL conical tube to purify conidia away from mycelia. One hundred microliters of conidia were used to inoculate 50 mL sterilized potato dextrose broth (PDB, Neogen) in a 250 mL flask. The flasks were incubated for 36–48 h at room temperature, with shaking at 125 rpm, to allow conidia to germinate.

Mycelia were collected aseptically using a sterile funnel and Miracloth (Millipore-Sigma), removing the spent PDB. Mycelia were transferred back into the empty 250 mL flask with a sterile spatula, and 30 mL of protoplasting solution was added immediately. The protoplasting solution was prepared one hour prior to protoplasting, composed of 1.2 M KCl, 750 mg driselase (Millipore-Sigma), 1.5 mg chitinase from *Streptomyces griseus* (Millipore-Sigma), and 150 mg lysing enzymes from *Trichoderma harzianum* (Millipore-Sigma) per 30 mL. The protoplasting solution was stirred for at least 30 min prior to use and filtered through a 0.45 µm filter just before adding to the mycelia. Once immersed in the protoplasting solution, the mycelia were incubated at 30 °C for 3–5 h with shaking at 75 rpm. A 10 µL aliquot of the protoplasting reaction was taken every 30–60 min to determine the level of protoplasts. After 3–5 h, the majority of the mycelia was visually determined to be digested and many protoplasts were easily identified with a microscope. The protoplasting reaction was filtered through a 30 µm Nylon mesh filter (Millipore-Sigma), with protoplasts passing through into a 50 mL conical tube. The protoplasts were centrifuged at 3200×*g* for 5 min at 4 °C, then gently resuspended in 10 mL of chilled STC buffer (1.2 M d-sorbitol, 10 mM CaCl_2_, 10 mM Tris–HCl, pH 7.5) using a wide orifice pipet tip (Mettler-Toledo Rainin, Oakland, CA). The centrifuge and resuspension steps were repeated twice, with the last resuspension using 1 mL of STC buffer. A subsample of the protoplasts was diluted 1:100 and quantified using a hemocytometer to determine the initial quantity. The protoplasts were diluted to a final concentration of 10^7^ per milliliter in STC buffer and transferred into aliquots of 400 µL. Dimethyl sulfoxide (DMSO) was added to a final concentration of 7%, and the protoplasts were stored immediately in a − 80 °C freezer.

### Transformation

**Fig. 2:**
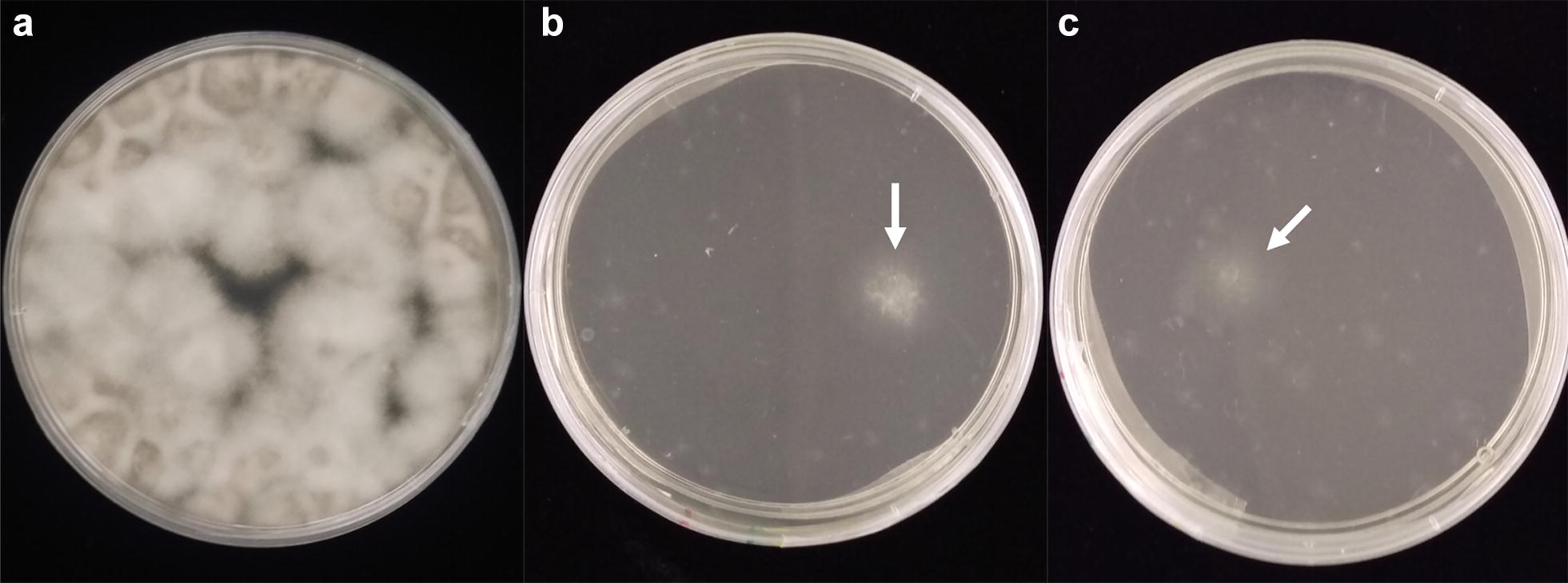
Representative protoplasts seven days post-transformation overlaid with non-amended regeneration medium (**a**), regeneration medium amended with hygromycin (**b**), or nourseothricin (**c**). Arrows represent putative transformants that have grown through the entire layer of amended medium

Protoplasts were removed from − 80 °C, thawed on ice, and centrifuged at 3000×*g* for 4 min at 4 °C. The supernatant was carefully pipetted out and the protoplasts were resuspended in 400 µL chilled STC buffer, to remove DMSO. The centrifugation and resuspension steps were repeated twice, with the final resuspension using 200 µL STC buffer. With the protoplasts on ice, 5 µg of linearized transformation vector was added, followed by 50 µL of 30% PEG-8000 (Sigma-Aldrich, Cat. No. P2139) solution (10 mM Tris–HCl, 50 mM CaCl_2_, 30% poly-ethylene glycol w/v), so that PEG comprised 20% of the transformation reaction volume. *Fusarium virguliforme* was transformed with 5 µg of a linear construct containing *hph* and eGFP, while *F. brasiliense* was transformed with 5 µg of a linear construct containing *nat* and mCherry (see maps in Additional file 2). The reactions were gently inverted 3 times, then incubated on ice for one hour. Using a wide orifice pipet tip, each reaction was transferred into a sterile 15 mL tube containing 2 mL 30% PEG-8000 and incubated for 15 min. Four milliliters of room-temperature STC buffer was added and gently inverted three times to mix. The reaction was then poured into 250 mL sterile, molten regeneration medium at 42 °C (67.75 g sucrose, 0.25 g yeast extract, 0.25 g N-Z amine, 1.86 g agar per 250 mL) [29]. The agar was swirled gently to mix, then poured as a thin layer into 20, 100 × 15 mm Petri plates (VWR International, Radnor, PA). After 24 h of incubation at room temperature, another layer of regeneration medium amended with either 100 µg/mL hygromycin B or 100 µg/mL nourseothricin was overlaid onto the germinating protoplasts. One plate was overlaid with non-amended regeneration medium as a control to determine viability of protoplasts after the transformation and plating procedures (Fig. 2).

### Screening for stable transformants

Putative transformants that grew through the layer of regeneration medium amended with antibiotics were transferred to PDA amended with antibiotics. After 10–14 days of growth, a single spore of the putative transformant was isolated and re-transferred to a new PDA plate amended with antibiotics. After 10–14 days of germination from this single spore, hyphae was scraped off the surface of the plate using a sterile inoculation loop and transferred onto a small pad of 2% agarose on a microscope slide, and viewed under a compound fluorescent microscope (Leica Microsystems, Buffalo Grove, IL). Stable transformants and wild type strains were viewed under bright field and fluorescent channels. To determine fluorescence of *F. virguliforme*, a GFP/FITC filter was used, and for *F. brasiliense* the TexasRed filter was used (Leica Microsystems).

## Results

*Fusarium virguliforme* is sensitive to hygromycin [9, 14, 15, 19, 23], but sensitivity to hygromycin has not been reported for *F. brasiliense*. Medium amended with 100 µg/mL hygromycin prevents growth in two wild type strains of *F. virguliforme* and *F. brasiliense* (Fig. 1). Another common antibiotic used for genetic selection is nourseothricin, which has been used in *F. graminearum* and *F. fujikuroi* transformations, but not *F. virguliforme* or *F. brasiliense* [30–32]. Medium amended with 100 µg/mL nourseothricin prevents growth in both wild type strains of *F. virguliforme* and *F. brasiliense* (Fig. 1). However, genetic mutants containing the antibiotic resistance genes *hph* or *nat* are able to grow on amended medium with hygromycin and nourseothricin, respectively. Representative growth of these mutants on amended medium is presented after 14 days (Fig. 1).

Following the protocol described in Additional file 4 and outlined in Additional file 5, high quality protoplasts of *F. virguliforme* were produced. The protocol was also successfully applied to *F. brasiliense*. These protoplasts were stored frozen at − 80 °C for up to two years and still showed viability throughout the transformation process (Fig. 2). Introducing the transformation vector to protoplasts resulted in successful transformation, which allowed growth on medium amended with a high concentration of the selectable antibiotic (Fig. 2).

**Fig. 3:**
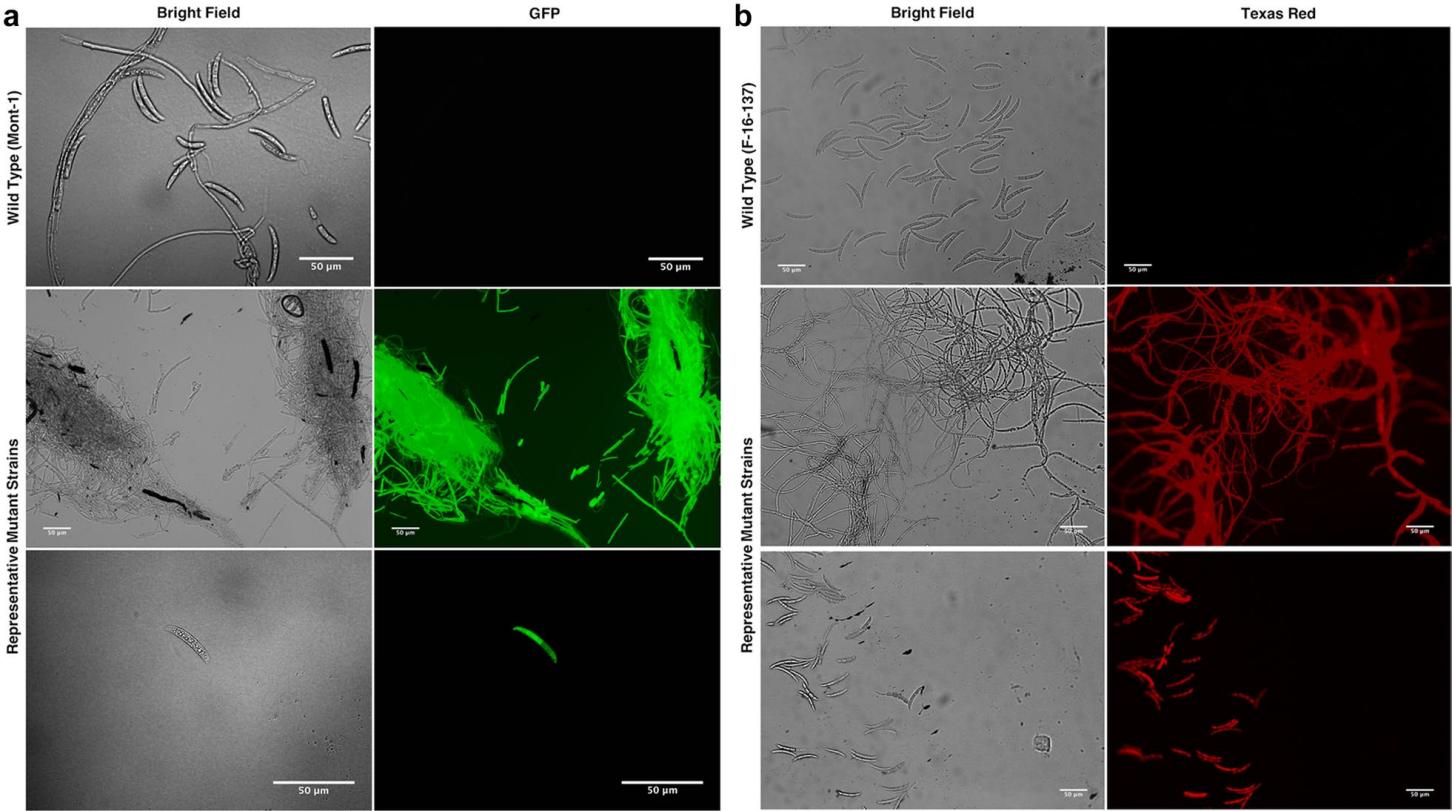
Microscopic features of wild type and representative mutant strains of *F. virguliforme* (**a**) and *F. brasiliense* (**b**). No autofluorescence was detected from the wild-type strains, but was detected in representative mutant strains transformed with a fluorescent reporter gene. All scale bars represent 50 µm

**Fig. 4:**
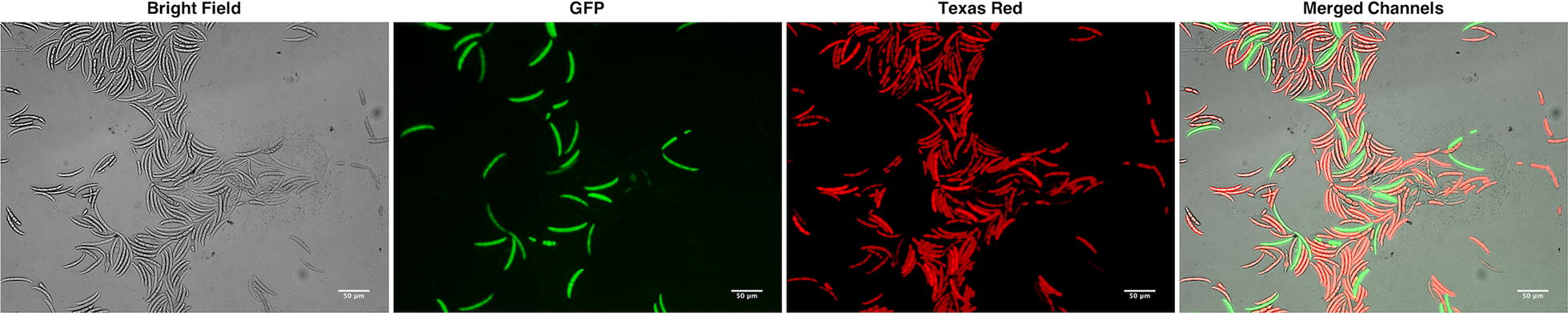
Mixed culture of *Fusarium* spores, distinguished by fluorescent reporter gene expression, with *F. virguliforme* fluorescing green and *F. brasiliense* fluorescing red under appropriate excitation spectra for eGFP and mCherry, respectively. All scale bars represent 50 µm

Beginning with 10^7^ protoplasts per transformation, up to 76 putative transformants were obtained in a single reaction (data not shown). Stable transformants were obtained and screened for fluorescence. Neither wild type strain of *F. virguliforme* or *F. brasiliense* demonstrated green or red autofluorescence (Fig. 3). However, many stable transformants displayed fluorescent phenotypes, and representative isolates are presented (Fig. 3). Phenotypic differentiation of *F. virguliforme* and *F. brasiliense* requires many spore measurements, which can be very challenging since spore lengths and widths overlap [2]. However, the identities of spores in a mixed culture the transgenic strains developed here are easily distinguished based on fluorescence (Fig. 4).

## Discussion

Generating protoplasts of *F. virguliforme* has been successful in other laboratories using a previously published protocol [9, 14, 15, 23, 24], but was unsuccessful in our hands. Protoplast generation was successful following the protocol developed for *F. graminearum*, but the transformation method reported therein was unsuccessful for transforming *F. virguliforme* and *F. brasiliense* [29]. The transformation protocol for *F. virguliforme*, as written, also failed to produce successful transformants for us [9]. Therefore, we integrated components from both of these published protocols, resulting in the protocol described here. Implementing this transformation protocol with high quality protoplasts allowed for the generation of stable transformants of both *F. virguliforme* and *F. brasiliense* (Fig. 2). In addition, *F. virguliforme* protoplasts developed with the method described here were successfully used in a targeted gene replacement study testing different fungal succinate dehydrogenase alleles [19].

The two previously published protoplasting protocols for *F. graminearum* and *F. virguliforme* differ in many aspects [9, 29]. These differences include buffer types, enzyme compositions, and enzyme concentrations. While the *F. virguliforme* protocol uses a sodium phosphate buffer containing β-glucuronidase and a high concentration of lysing enzymes (25 mg/mL), the *F. graminearum* protocol uses a potassium chloride buffer with chitinase rather than β-glucuronidase, and a lower lysing enzyme concentration (5 mg/mL). Given that chitin and β-glucan are the major polysaccharides found in the cell walls of *Fusarium* spp., the addition of chitinase likely increased our protoplasting efficiency for *F. virguliforme* and *F. brasiliense* [33]. It is worth noting that 3–5 h were required to gain sufficient protoplasts in *F. virguliforme* and *F. brasiliense* compared to the 2 h reported for *F. graminearum* [29]. A detailed comparison between protoplasting buffers and enzyme compositions also concluded that potassium chloride was optimal for protoplast generation in the fungal pathogen *F. verticillioides*, and the addition of chitinase helps generate protoplasts in other *Fusarium* species [34].

*Fusarium brasiliense* is a close relative of *F. virguliforme*, as both are members of clade II in the *Fusarium solani* species complex [2, 6]. However, different members of this species complex display different sensitivities to fluopyram, a common fungicide used to control these species [19]. Understanding the genetic mechanisms of fungicide sensitivity or resistance can be elucidated by testing gene function through gene knockout and gene replacement studies using split marker gene replacement constructs [35]. The genetic mechanisms of SDS development are poorly characterized, and whether mechanisms of SDS development are shared between *F. virguliforme* and *F. brasiliense* are also unknown. Though we introduced foreign DNA into *F. brasiliense* via random insertion in this study, this protocol should also facilitate knock out experiments via gene replacement as it has in *F. virguliforme* protoplasts obtained with this method [19].

Both *F. virguliforme* and *F. brasiliense* are capable of infecting soybean and common bean (*Phaseolus vulgaris*) [8, 10, 36], and isolation frequency data suggest that *F. brasiliense* may have a host preference for common bean [8]. The development of transgenic *F. virguliforme* and *F. brasiliense* strains expressing different fluorescent markers may allow for studies investigating host preference through co-inoculations, as they can easily be distinguished under the microscope based on their reporter gene expression (Fig. 4). With this protocol and future experiments we can gain a greater genetic understanding of these pathogens which could lead to new and effective management strategies.

## Conclusions

*Fusarium virguliforme* and *F. brasiliense* are both causal agents of soybean SDS and are amenable to genetic manipulation for studying gene function. Genetic transformation in these species requires high quality protoplasts. The protocol developed here provides methods for producing high quality protoplasts that builds upon previously reported protocols and should be valuable to the soybean SDS and common bean root rot research community. The fluorescent strains developed here can be used to investigate temporal colonization and co-infections of plant roots as well as the potential host preferences of these species. Using this protocol to probe gene function in these pathogens will be critical to understanding their pathology and advancing plant genetics to improve SDS management.

## Additional files

**Additional file 1.** A supplementary table describing the primers used in this study.

**Additional file 2.** A supplementary figure showing maps of plasmids developed in this study.

**Additional file 3.** A supplementary file reporting the sequence of the plasmids developed in this study, in FASTA format.

**Additional file 4.** A supplementary file presenting a step-by-step protocol for protoplasting and transforming *Fusarium virguliforme* and *Fusarium brasiliense.*

**Additional file 5.** A supplementary figure presenting an overall depiction of the protoplasting and transformation steps.

## References

[1] Allen TW, Bradley CA, Sisson AJ, Byamukama E, Chilvers MI, Coker CM (2017). Soybean yield loss estimates due to diseases in the United States and Ontario, Canada, from 2010 to 2014. Plant Heal Prog.

[2] Aoki T, O’Donnell K, Scandiani MM (2005). Sudden death syndrome of soybean in South America is caused by four species of *Fusarium*: *Fusarium brasiliense* sp. nov., *F cuneirosrum* sp. nov., *F. tucumaniae*, and *F. virguliforme*. Mycoscience.

[3] Tewoldemedhin YT, Lamprecht SC, Geldenhuys JJ, Kloppers FJ (2014). First report of soybean sudden death syndrome caused by *Fusarium virguliforme* in South Africa. Plant Dis.

[4] Tewoldemedhin YT, Lamprecht SC, Vaughan MM, Doehring G, O’Donnell K (2016). Soybean SDS in South Africa is caused by *Fusarium brasiliense* and a novel undescribed *Fusarium* sp. Plant Dis.

[5] Aoki T, O’Donnell K, Geiser DM (2014). Systematics of key phytopathogenic *Fusarium* species: current status and future challenges. J Gen Plant Pathol.

[6] Chitrampalam P, Nelson B (2016). Multilocus phylogeny reveals an association of agriculturally important *Fusarium solani* species complex (FSSC) 11, and clinically important FSSC 5 and FSSC 3 + 4 with soybean roots in the north central United States. Antonie van Leeuwenhoek.

[7] Roy KW, Hershman DE, Rupe JC, Abney TS (1997). Sudden death syndrome of soybean. Plant Dis.

[8] Wang J, Sang H, Jacobs JL, Oudman KA, Hanson LE, Chilvers MI (2018). Soybean sudden death syndrome causal agent *Fusarium brasiliense* present in Michigan. Plant Dis.

[9] Mansouri S, Van Wijk R, Rep M, Fakhoury AM (2009). Transformation of *Fusarium virguliforme*, the causal agent of sudden death syndrome of soybean. J Phytopathol.

[10] Jacobs JL, Oudman K, Sang H, Chilvers MI (2018). First report of *Fusarium brasiliense* causing root rot of dry bean in the United States. Plant Dis.

[11] Chang H-X, Yendrek CR, Caetano-Anolles G, Hartman GL (2016). Genomic characterization of plant cell wall degrading enzymes and in silico analysis of xylanses and polygalacturonases of *Fusarium virguliforme*. BMC Microbiol.

[12] Navi SS, Yang XB (2008). Foliar symptom expression in association with early infection and xylem colonization by *Fusarium virguliforme* (formerly *F. solani* f. sp. *glycines*), the causal agent of soybean sudden death syndrome. Plant Heal Prog.

[13] Brar HK, Swaminathan S, Bhattacharyya MK (2011). The *Fusarium virguliforme* toxin FvTox1 causes foliar sudden death syndrome-like symptoms in soybean. Mol Plant Microbe Interact.

[14] Chang H-X, Domier LL, Radwan O, Yendrek CR, Hudson ME, Hartman GL (2016). Identification of multiple phytotoxins produced by *Fusarium virguliforme* including a phytotoxic effector (FvNIS1) associated with sudden death syndrome foliar symptoms. Mol Plant Microbe Interact.

[15] Pudake RN, Swaminathan S, Sahu BB, Leandro LF, Bhattacharyya MK (2013). Investigation of the *Fusarium virguliforme fvtox1* mutants revealed that the FvTox1 toxin is involved in foliar sudden death syndrome development in soybean. Curr Genet.

[16] Hartman GL, Chang H-X, Leandro LF (2015). Research advances and management of soybean sudden death syndrome. Crop Prot.

[17] Kandel YR, Mueller DS, Legleiter T, Johnson WG, Young BG, Wise KA (2018). Impact of fluopyram fungicide and preemergence herbicides on soybean injury, population, sudden death syndrome, and yield. Crop Prot.

[18] Leandro LFS, Eggenberger S, Chen C, Williams J, Beattie GA, Liebman M (2018). Cropping system diversification reduces severity and incidence of soybean sudden death syndrome caused by *Fusarium virguliforme*. Plant Dis.

[19] Sang H, Witte A, Jacobs JL, Chang H-X, Wang J, Roth MG (2018). Fluopyram sensitivity and functional characterization of SdhB in the *Fusarium solani* species complex causing soybean sudden death syndrome. Front Microbiol.

[20] Huang X, Das A, Sahu BB, Srivastava SK, Leandro LF, O’Donnell K, Bhattacharyya MK (2016). Identification of highly variable supernumerary chromosome segments in an asexual pathogen. PLoS ONE.

[21] Srivastava SK, Huang X, Brar HK, Fakhoury AM, Bluhm BH, Bhattacharyya MK (2016). The genome sequence of the fungal pathogen *Fusarium virguliforme* that causes sudden death syndrome in soybean. PLoS ONE.

[22] Peberdy JF (1979). Fungal protoplasts: isolation, reversion, and fusion. Annu Rev Microbiol.

[23] Islam KT, Bond JP, Fakhoury AM (2017). FvSTR1, a striatin orthologue in *Fusarium virguliforme*, is required for asexual development and virulence. Appl Microbiol Biotechnol.

[24] Islam KT, Bond JP, Fakhoury AM (2017). FvSNF1, the sucrose non-fermenting protein kinase gene of *Fusarium virguliforme*, is required for cell-wall-degrading enzymes expression and sudden death syndrome development in soybean. Curr Genet.

[25] Gibson DG, Young L, Chuang R-Y, Venter JC, Hutchison CA, Smith HO (2009). Enzymatic assembly of DNA molecules up to several hundred kilobases. Nat Methods.

[26] MacCready JS, Schossau J, Osteryoung KW, Ducat DC (2017). Robust Min-system oscillation in the presence of internal photosynthetic membranes in cyanobacteria. Mol Microbiol.

[27] Carroll AM, Sweigard JA, Valent B (1994). Improved vectors for selecting resistance to hygromycin. Fungal Genet Newsl.

[28] Ivanov S, Harrison MJ (2014). A set of fluorescent protein-based markers expressed from constitutive and arbuscular mycorrhiza-inducible promoters to label organelles, membranes and cytoskeletal elements in *Medicago truncatula*. Plant J.

[29] Hallen-Adams HE, Cavinder BL, Trail F, Xu JR, Bluhm BH (2011). *Fusarium graminearum* from expression analysis to functional assays. Fungal genomics.

[30] Menke J, Weber J, Broz K, Kistler HC (2013). Cellular development associated with induced mycotoxin synthesis in the filamentous fungus *Fusarium graminearum*. PLoS ONE.

[31] Wiemann P, Willmann A, Straeten M, Kleigrewe K, Beyer M, Humpf HU (2009). Biosynthesis of the red pigment bikaverin in *Fusarium fujikuroi*: genes, their function and regulation. Mol Microbiol.

[32] Wiemann P, Brown DW, Kleigrewe K, Bok JW, Keller NP, Humpf HU (2010). FfVel1 and fflae1, components of a velvet-like complex in *Fusarium fujikuroi*, affect differentiation, secondary metabolism and virulence. Mol Microbiol.

[33] Schoffelmeer EAM, Klis FM, Sietsma JH, Cornelissen BJC (1999). The cell wall of *Fusarium oxysporum*. Fungal Genet Biol.

[34] Ramamoorthy V, Govindaraj L, Dhanasekaran M, Vetrivel S, Kumar KK, Ebenezar E (2015). Combination of driselase and lysing enzyme in one molar potassium chloride is effective for the production of protoplasts from germinated conidia of *Fusarium verticillioides*. J Microbiol Methods.

[35] Catlett NL, Lee B-N, Yoder OC, Turgeon BG (2003). Split-marker recombination for efficient targeted deletion of fungal genes. Fungal Genet Newsl.

[36] Kolander TM, Bienapfl JC, Kurle JE, Malvick DK (2012). Symptomatic and asymptomatic host range of *Fusarium virguliforme*, the causal agent of soybean sudden death syndrome. Plant Dis.

