# Supplementary material for: A protoplast generation and transformation method for soybean sudden death syndrome causal agents Fusarium virguliforme and F. brasiliense
Source: Fungal Biol Biotechnol. 2019 May 15;6:7. doi: 10.1186/s40694-019-0070-0 (PMC6518667; doi:10.1186/s40694-019-0070-0)
Supplement: Supplementary file 1 — Additional file 1. A supplementary table describing the primers used in this study. [file 40694_2019_70_MOESM1_ESM.docx]

| **Primer name** | **Sequence (5’-3’)** | **Template** | **PCR Product Length** | **Purpose** |
| --- | --- | --- | --- | --- |
| Nat_F_NheI | GACGACAGCTAGCATCGATCTGACGATACTTTCTAGAGAATAGGAACTTCGG | pDS23-eGFP | 1333 bp | Amplify *nat* gene |
| Nat_R_EcoRI | AGTTCTGGTGAATTCTCACCAGTGTAACTGATATTGAAGGAGCATTTTTTGG |  |  |  |
| HindIII-mChF | GTGTGAAGCTTATGGTGAGCAAGGGCGAG | pCMB-TMeR | 708 bp | Cloning mCherry into pDS23-eGFP |
| NotI-mChR | TTCTTGCGGCCGCCTACTTGTACAGCTCGTCCATGCC |  |  |  |
| GPD_F_PstI | GACGACACTGCAGATCGATCTGACGGTACAGTGACCGGTGACTCTTTCTGG | pDS23-eGFP | 2410 / 2427 bp | Amplifying GPD promoter + eGFP + TrpC terminator |
| GPD_F_SacI | ATGCTCGATGAGTTTTTCTAAGAGCTCTGTACAGTGACCGGTGACT | pDS23-eGFP with eGFP replaced by mCherry | 2410 bp | Amplifying GPD promoter + mCherry + TrpC terminator |
| TrpC_R_NheI | AGATCGATGCTAGCTGTCGTCGACTTCGAGTGGAGATGTGGAGTG | Both versions of pDS23 (eGFP or mCherry) | 2427 bp | Amplifying GPD promoter + eGFP or mCherry + TrpC terminator |
| OriF_EcoRI | TGGTGAGAATTCACCAGAACTGTCAAGATCAAAGGATCTTCTTGAGATCCTT | pJM2016 | 655 bp | Amplify high copy number OriC |
| Ori-F2 | TGGCTTTCCCCGCGTTGCTGGCGTT |  |  |  |
| KanF3 | GCCAGCAACGCGGGGAAAGCCACGTTGTGTCTC | pJM2016 | 941 bp | Amplify kanamycin resistance gene |
| Kan-R | TTAGAAAAACTCATCGAGCATCAAATGAAACTGCA |  |  |  |

**Table S1.** Primer pairs designed and used in this study for cloning and assembly into plasmids.
