# Supplementary figures and images for: A protoplast generation and transformation method for soybean sudden death syndrome causal agents Fusarium virguliforme and F. brasiliense
Source: Fungal Biol Biotechnol. 2019 May 15;6:7. doi: 10.1186/s40694-019-0070-0 (PMC6518667; doi:10.1186/s40694-019-0070-0)

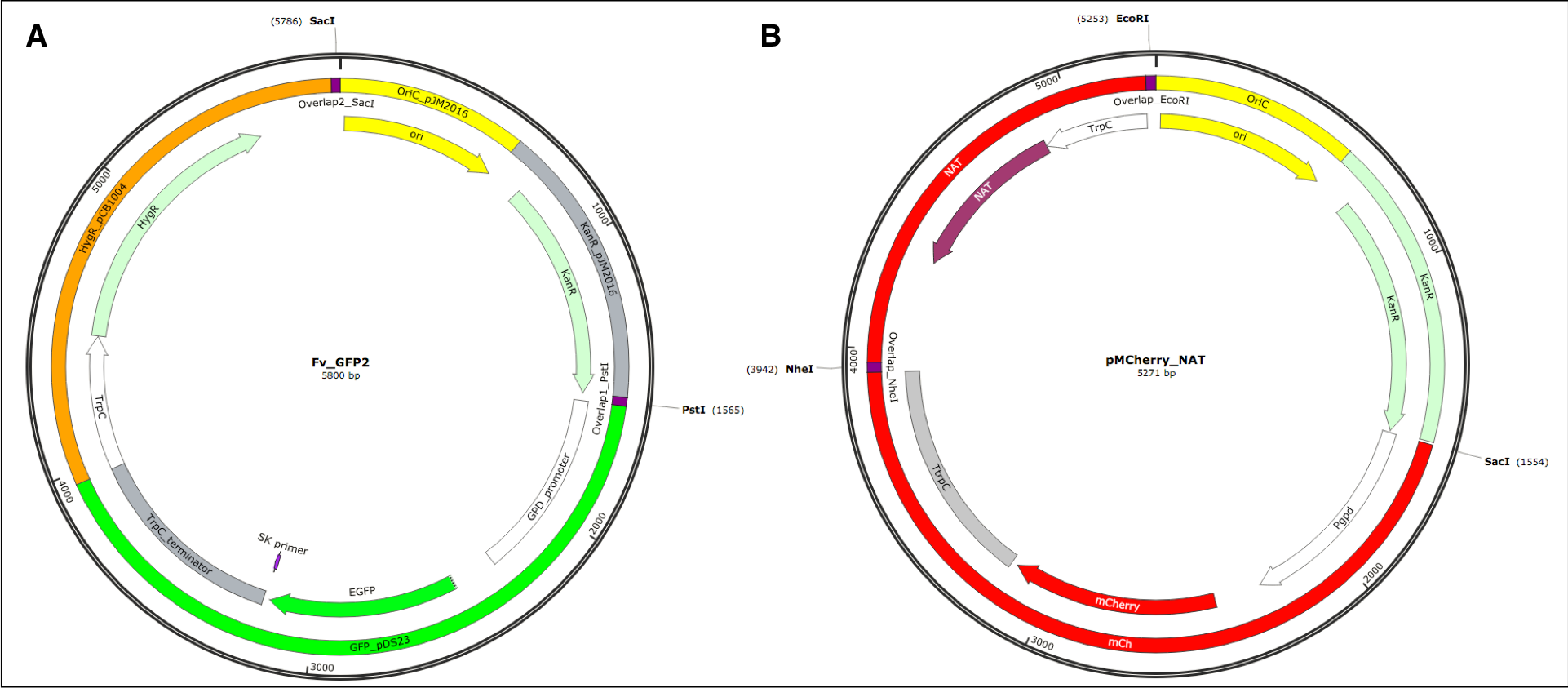

Supplement: Supplementary file 2 — Additional file 2. A supplementary figure showing maps of plasmids developed in this study. [file 40694_2019_70_MOESM2_ESM.tiff]

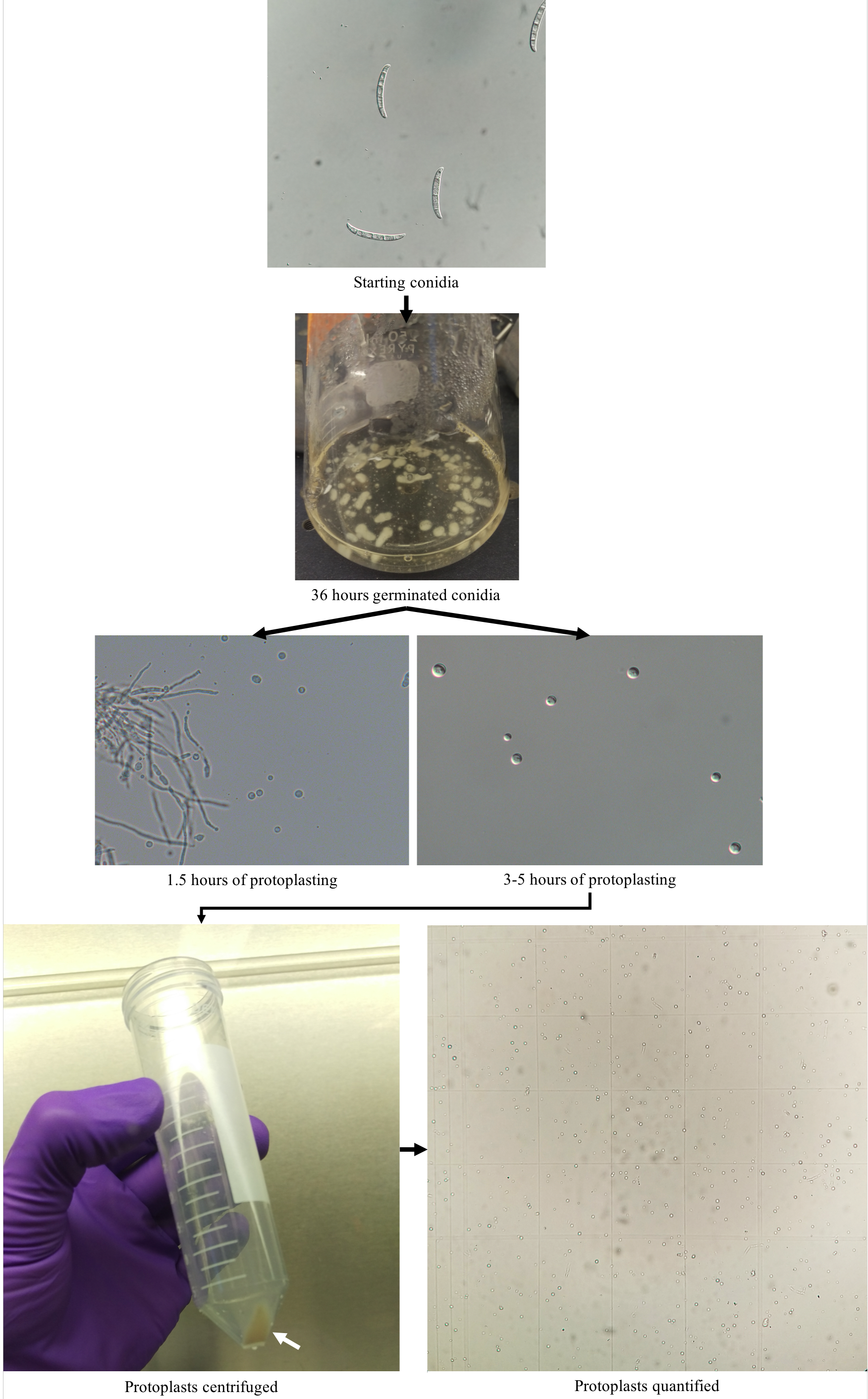

Supplement: Supplementary file 5 — Additional file 5. A supplementary figure presenting an overall depiction of the protoplasting and transformation steps. [file 40694_2019_70_MOESM5_ESM.tiff]
