# Supplementary material for: A protoplast generation and transformation method for soybean sudden death syndrome causal agents Fusarium virguliforme and F. brasiliense
Source: Fungal Biol Biotechnol. 2019 May 15;6:7. doi: 10.1186/s40694-019-0070-0 (PMC6518667; doi:10.1186/s40694-019-0070-0)
Supplement: Supplementary file 3 — Additional file 3. A supplementary file reporting the sequence of the plasmids developed in this study, in FASTA format. [file 40694_2019_70_MOESM3_ESM.docx]

>Fv_GFP2

AGATCAAAGGATCTTCTTGAGATCCTTTTTTTCTGCGCGTAATCTGCTGCTTGCAAACAAAAAAACCACCGCTACCAGCGGTGGTTTGTTTGCCGGATCAAGAGCTACCAACTCTTTTTCCGAAGGTAACTGGCTTCAGCAGAGCGCAGATACCAAATACTGTTCTTCTAGTGTAGCCGTAGTTAGGCCACCACTTCAAGAACTCTGTAGCACCGCCTACATACCTCGCTCTGCTAATCCTGTTACCAGTGGCTGCTGCCAGTGGCGATAAGTCGTGTCTTACCGGGTTGGACTCAAGACGATAGTTACCGGATAAGGCGCAGCGGTCGGGCTGAACGGGGGGTTCGTGCACACAGCCCAGCTTGGAGCGAACGACCTACACCGAACTGAGATACCTACAGCGTGAGCTATGAGAAAGCGCCACGCTTCCCGAAGGGAGAAAGGCGGACAGGTATCCGGTAAGCGGCAGGGTCGGAACAGGAGAGCGCACGAGGGAGCTTCCAGGGGGAAACGCCTGGTATCTTTATAGTCCTGTCGGGTTTCGCCACCTCTGACTTGAGCGTCGATTTTTGTGATGCTCGTCAGGGGGGCGGAGCCTATGGAAAAACGCCAGCAACGCGGGGAAAGCCACGTTGTGTCTCAAAATCTCTGATGTTACATTGCACAAGATAAAAATATATCATCATGAACAATAAAACTGTCTGCTTACATAAACAGTAATACAAGGGGTGTTATGAGCCATATTCAACGGGAAACGTCTTGCTCGAGGCCGCGATTAAATTCCAACATGGATGCTGATTTATATGGGTATAAATGGGCTCGCGATAATGTCGGGCAATCAGGTGCGACAATCTATCGATTGTATGGGAAGCCCGATGCGCCAGAGTTGTTTCTGAAACATGGCAAAGGTAGCGTTGCCAATGATGTTACAGATGAGATGGTCAGACTAAACTGGCTGACGGAATTTATGCCTCTTCCGACCATCAAGCATTTTATCCGTACTCCTGATGATGCATGGTTACTCACCACTGCGATCCCCGGGAAAACAGCATTCCAGGTATTAGAAGAATATCCTGATTCAGGTGAAAATATTGTTGATGCGCTGGCAGTGTTCCTGCGCCGGTTGCATTCGATTCCTGTTTGTAATTGTCCTTTTAACAGCGATCGCGTATTTCGTCTCGCTCAGGCGCAATCACGAATGAATAACGGTTTGGTTGATGCGAGTGATTTTGATGACGAGCGTAATGGCTGGCCTGTTGAACAAGTCTGGAAAGAAATGCATAAGCTTTTGCCATTCTCACCGGATTCAGTCGTCACTCATGGTGATTTCTCACTTGATAACCTTATTTTTGACGAGGGGAAATTAATAGGTTGTATTGATGTTGGACGAGTCGGAATCGCAGACCGATACCAGGATCTTGCCATCCTATGGAACTGCCTCGGTGAGTTTTCTCCTTCATTACAGAAACGGCTTTTTCAAAAATATGGTATTGATAATCCTGATATGAATAAATTGCAGTTTCATTTGATGCTCGATGAGTTTTTCTAAAGTCGACGACACTGCAGATCGATCTGACGGTACAGTGACCGGTGACTCTTTCTGGCATGCGGAGAGACGGACGGACGCAGAGAGAAGGGCTGAGTAATAAGCGCCACTGCGCCAGACAGCTCTGGCGGCTCTGAGGTGCAGTGGATGATTATTAATCCGGGACCGGCCGCCCCTCCGCCCCGAAGTGGAAAGGCTGGTGTGCCCCTCGTTGACCAAGAATCTATTGCATCATCGGAGAATATGGAGCTTCATCGAATCACCGGCAGTAAGCGAAGGAGAATGTGAAGCCAGGGGTGTATAGCCGTCGGCGAAATAGCATGCCATTAACCTAGGTACAGAAGTCCAATTGCTTCCGATCTGGTAAAAGATTCACGAGATAGTACCTTCTCCGAAGTAGGTAGAGCGAGTACCCGGCGCGTAAGCTCCCTAATTGGCCCATCCGGCATCTGTAGGGCGTCCAAATATCGTGCCTCTCCTGCTTTGCCCGGTGTATGAAACCGGAAAGGCCGCTCAGGAGCTGGCCAGCGGCGCAGACCGGGAACACAAGCTGGCAGTCGACCCATCCGGTGCTCTGCACTCGACCTGCTGAGGTCCCTCAGTCCCTGGTAGGCAGCTTTGCCCCGTCTGTCCGCCCGGTGTGTCGGCGGGGTTGACAAGGTCGTTGCGTCAGTCCAACATTTGTTGCCATATTTTCCTGCTCTCCCCACCAGCTGCTCTTTTCTTTTCTCTTTCTTTTCCCATCTTCAGTATATTCATCTTCCCATCCAAGAACCTTTATTTCCCCTAAGTAAGTACTTTGCTACATCCATACTCCATCCTTCCCATCCCTTATTCCTTTGAACCTTTCAGTTCGAGCTTTCCCACTTCATCGCAGCTTGACTAACAGCTACAGATCTAAGCTTATGGTGAGCAAGGGCGAGGAGCTGTTCACCGGGGTGGTGCCCATCCTGGTCGAGCTGGACGGCGACGTAAACGGCCACAAGTTCAGCGTGTCCGGCGAGGGCGAGGGCGATGCCACCTACGGCAAGCTGACCCTGAAGTTCATCTGCACCACCGGCAAGCTGCCCGTGCCCTGGCCCACCCTCGTGACCACCCTGACCTACGGCGTGCAGTGCTTCAGCCGCTACCCCGACCACATGAAGCAGCACGACTTCTTCAAGTCCGCCATGCCCGAAGGCTACGTCCAGGAGCGCACCATCTTCTTCAAGGACGACGGCAACTACAAGACCCGCGCCGAGGTGAAGTTCGAGGGCGACACCCTGGTGAACCGCATCGAGCTGAAGGGCATCGACTTCAAGGAGGACGGCAACATCCTGGGGCACAAGCTGGAGTACAACTACAACAGCCACAACGTCTATATCATGGCCGACAAGCAGAAGAACGGCATCAAGGTGAACTTCAAGATCCGCCACAACATCGAGGACGGCAGCGTGCAGCTCGCCGACCACTACCAGCAGAACACCCCCATCGGCGACGGCCCCGTGCTGCTGCCCGACAACCACTACCTGAGCACCCAGTCCGCCCTGAGCAAAGACCCCAACGAGAAGCGCGATCACATGGTCCTGCTGGAGTTCGTGACCGCCGCCGGGATCACTCTCGGCATGGACGAGCTGTACAAGTAAAGCGGCCGCTCTAGAACTAGTGGATCCACTTAACGTTACTGAAATCATCAAACAGCTTGACGAATCTGGATATAAGATCGTTGGTGTCGATGTCAGCTCCGGAGTTGAGACAAATGGTGTTCAGGATCTCGATAAGATACGTTCATTTGTCCAAGCAGCAAAGAGTGCCTTCTAGTGATTTAATAGCTCCATGTCAACAAGAATAAAACGCGTTTTCGGGTTTACCTCTTCCAGATACAGCTCATCTGCAATGCATTAATGCATTGACTGCAACCTAGTAACGCCTTNCAGGCTCCGGCGAAGAGAAGAATAGCTTAGCAGAGCTATTTTCATTTTCGGGAGACGAGATCAAGCAGATCAACGGTCGTCAAGAGACCTACGAGACTGAGGAATCCGCTCTTGGCTCCACGCGACTATATATTTGTCTCTAATTGTACTTTGACATGCTCCTCTTCTTTACTCTGATAGCTTGACTATGAAAATTCCGTCACCAGCNCCTGGGTTCGCAAAGATAATTGCATGTTTCTTCCTTGAACTCTCAAGCCTACAGGACACACATTCATCGTAGGTATAAACCTCGAAATCANTTCCTACTAAGATGGTATACAATAGTAACCATGCATGGTTGCCTAGTGAATGCTCCGTAACACCCAATACGCCGGCCGAAACTTTTTTACAACTCTCCTATGAGTCGTTTACCCAGAATGCACAGGTACACTTGTTTAGAGGTAATCCTTCTTTCTAGAAGTCCTCGTGTACTGTGTAAGCGCCCACTCCACATCTCCACTCGAAACGCTGGTGAAAGTAAAAGATGCTGAAGATCAGTTGGGTGCACGAGTGGGTTACATCGAACTGGATCTCAACAGCGGTAAGATCCTTGAGAGTTTTCGCCGCGAAGAACGTTTTCCAATGATGAGCACTTTTTGATCGACGTTAACTGATATTGAAGGAGCATTTTTTGGGCTTGGCTGGAGCTAGTGGAGGTCAACAATGAATGCCTATTTTGGTTTAGTCGTCCAGGCGGTGAGCACAAAATTTGTGTCGTTTGACAAGATGGTTCATTTAGGCAACTGGTCAGATCAGCCCCACTTGTAGCAGTAGCGGCGGCGCTCGAAGTGTGACTCTTATTAGCAGACAGGAACGAGGACATTATTATCATCTGCTGCTTGGTGCACGATAACTTGGTGCGTTTGTCAAGCAAGGTAAGTGGACGACCCGGTCATACCTTCTTAAGTTCGCCCTTCCTCCCTTTATTTCAGATTCAATCTGACTTACCTATTCTACCCAAGCATCCAAATGAAAAAGCCTGAACTCACCGCGACGTCTGTCGAGAAGTTTCTGATCGAAAAGTTCGACAGCGTCTCCGACCTGATGCAGCTCTCGGAGGGCGAAGAATCTCGTGCTTTCAGCTTCGATGTAGGAGGGCGTGGATATGTCCTGCGGGTAAATAGCTGCGCCGATGGTTTCTACAAAGATCGTTATGTTTATCGGCACTTTGCATCGGCCGCGCTCCCGATTCCGGAAGTGCTTGACATTGGGGAGTTCAGCGAGAGCCTGACCTATTGCATCTCCCGCCGTGCACAGGGTGTCACGTTGCAAGACCTGCCTGAAACCGAACTGCCCGCTGTTCTCCAGCCGGTCGCGGAGGCCATGGATGCGATCGCTGCGGCCGATCTTAGCCAGACGAGCGGGTTCGGCCCATTCGGACCGCAAGGAATCGGTCAATACACTACATGGCGTGATTTCATATGCGCGATTGCTGATCCCCATGTGTATCACTGGCAAACTGTGATGGACGACACCGTCAGTGCGTCCGTCGCGCAGGCTCTCGATGAGCTGATGCTTTGGGCCGAGGACTGCCCCGAAGTCCGGCACCTCGTGCATGCGGATTTCGGCTCCAACAATGTCCTGACGGACAATGGCCGCATAACAGCGGTCATTGACTGGAGCGAGGCGATGTTCGGGGATTCCCAATACGAGGTCGCCAACATCCTCTTCTGGAGGCCGTGGTTGGCTTGTATGGAGCAGCAGACGCGCTACTTCGAGCGGAGGCATCCGGAGCTTGCAGGATCGCCGCGCCTCCGGGCGTATATGCTCCGCATTGGTCTTGACCAACTCTATCAGAGCTTGGTTGACGGCAATTTCGATGATGCAGCTTGGGCGCAGGGTCGATGCGACGCAATCGTCCGATCCGGAGCCGGGACTGTCGGGCGTACACAAATCGCCCGCAGAAGCGCGGCCGTCTGGACCGATGGCTGTGTAGAAGTACTCGCCGATAGTGGAAACCGACGCCCCAGCACTCGTCCGAGGGCAAAGGAATAGAGTAGATGCCGACCGGGAACCAGTTAACGTCGATCAGAAAAAAAGGATCATATCGTCAATTATTACCTCCACGGGGAGAGCCTGAGCAAACTGGCCTCAGGCATTTGAGAAGCACACGGTCACACTGCTTCCGGTAGTCAATAAACCGGTAAACCAGCAATAGACATAAGCGGCTATTTAACGACCCTGCCCTGAACCGACGACCGGGTCGAATTTGCTTTCGAATTTCTGCCATTCATCCGCTTATTATCACTTATTCAGGCGTAGCAACCAGGCGTACACTGGTGAGAGCTCACCAGAACTGTCA

>pMCherry_NAT

AGATCAAAGGATCTTCTTGAGATCCTTTTTTTCTGCGCGTAATCTGCTGCTTGCAAACAAAAAAACCACCGCTACCAGCGGTGGTTTGTTTGCCGGATCAAGAGCTACCAACTCTTTTTCCGAAGGTAACTGGCTTCAGCAGAGCGCAGATACCAAATACTGTTCTTCTAGTGTAGCCGTAGTTAGGCCACCACTTCAAGAACTCTGTAGCACCGCCTACATACCTCGCTCTGCTAATCCTGTTACCAGTGGCTGCTGCCAGTGGCGATAAGTCGTGTCTTACCGGGTTGGACTCAAGACGATAGTTACCGGATAAGGCGCAGCGGTCGGGCTGAACGGGGGGTTCGTGCACACAGCCCAGCTTGGAGCGAACGACCTACACCGAACTGAGATACCTACAGCGTGAGCTATGAGAAAGCGCCACGCTTCCCGAAGGGAGAAAGGCGGACAGGTATCCGGTAAGCGGCAGGGTCGGAACAGGAGAGCGCACGAGGGAGCTTCCAGGGGGAAACGCCTGGTATCTTTATAGTCCTGTCGGGTTTCGCCACCTCTGACTTGAGCGTCGATTTTTGTGATGCTCGTCAGGGGGGCGGAGCCTATGGAAAAACGCCAGCAACGCGGGGAAAGCCACGTTGTGTCTCAAAATCTCTGATGTTACATTGCACAAGATAAAAATATATCATCATGAACAATAAAACTGTCTGCTTACATAAACAGTAATACAAGGGGTGTTATGAGCCATATTCAACGGGAAACGTCTTGCTCGAGGCCGCGATTAAATTCCAACATGGATGCTGATTTATATGGGTATAAATGGGCTCGCGATAATGTCGGGCAATCAGGTGCGACAATCTATCGATTGTATGGGAAGCCCGATGCGCCAGAGTTGTTTCTGAAACATGGCAAAGGTAGCGTTGCCAATGATGTTACAGATGAGATGGTCAGACTAAACTGGCTGACGGAATTTATGCCTCTTCCGACCATCAAGCATTTTATCCGTACTCCTGATGATGCATGGTTACTCACCACTGCGATCCCCGGGAAAACAGCATTCCAGGTATTAGAAGAATATCCTGATTCAGGTGAAAATATTGTTGATGCGCTGGCAGTGTTCCTGCGCCGGTTGCATTCGATTCCTGTTTGTAATTGTCCTTTTAACAGCGATCGCGTATTTCGTCTCGCTCAGGCGCAATCACGAATGAATAACGGTTTGGTTGATGCGAGTGATTTTGATGACGAGCGTAATGGCTGGCCTGTTGAACAAGTCTGGAAAGAAATGCATAAGCTTTTGCCATTCTCACCGGATTCAGTCGTCACTCATGGTGATTTCTCACTTGATAACCTTATTTTTGACGAGGGGAAATTAATAGGTTGTATTGATGTTGGACGAGTCGGAATCGCAGACCGATACCAGGATCTTGCCATCCTATGGAACTGCCTCGGTGAGTTTTCTCCTTCATTACAGAAACGGCTTTTTCAAAAATATGGTATTGATAATCCTGATATGAATAAATTGCAGTTTCATTTGATGCTCGATGAGTTTTTCTAAGAGCTCTGTACAGTGACCGGTGACTCTTTCTGGCATGCGGAGAGACGGACGGACGCAGAGAGAAGGGCTGAGTAATAAGCGCCACTGCGCCAGACAGCTCTGGCGGCTCTGAGGTGCAGTGGATGATTATTAATCCGGGACCGGCCGCCCCTCCGCCCCGAAGTGGAAAGGCTGGTGTGCCCCTCGTTGACCAAGAATCTATTGCATCATCGGAGAATATGGAGCTTCATCGAATCACCGGCAGTAAGCGAAGGAGAATGTGAAGCCAGGGGTGTATAGCCGTCGGCGAAATAGCATGCCATTAACCTAGGTACAGAAGTCCAATTGCTTCCGATCTGGTAAAAGATTCACGAGATAGTACCTTCTCCGAAGTAGGTAGAGCGAGTACCCGGCGCGTAAGCTCCCTAATTGGCCCATCCGGCATCTGTAGGGCGTCCAAATATCGTGCCTCTCCTGCTTTGCCCGGTGTATGAAACCGGAAAGGCCGCTCAGGAGCTGGCCAGCGGCGCAGACCGGGAACACAAGCTGGCAGTCGACCCATCCGGTGCTCTGCACTCGACCTGCTGAGGTCCCTCAGTCCCTGGTAGGCAGCTTTGCCCCGTCTGTCCGCCCGGTGTGTCGGCGGGGTTGACAAGGTCGTTGCGTCAGTCCAACATTTGTTGCCATATTTTCCTGCTCTCCCCACCAGCTGCTCTTTTCTTTTCTCTTTCTTTTCCCATCTTCAGTATATTCATCTTCCCATCCAAGAACCTTTATTTCCCCTAAGTAAGTACTTTGCTACATCCATACTCCATCCTTCCCATCCCTTATTCCTTTGAACCTTTCAGTTCGAGCTTTCCCACTTCATCGCAGCTTGACTAACAGCTACAGATCTAAGCTTATGGTGAGCAAGGGCGAGGAGGATAACATGGCCATCATCAAGGAGTTCATGCGCTTCAAGGTGCACATGGAGGGCTCCGTGAACGGCCACGAGTTCGAGATCGAGGGCGAGGGCGAGGGCCGCCCCTACGAGGGCACCCAGACCGCCAAGCTGAAGGTGACCAAGGGTGGCCCCCTGCCCTTCGCCTGGGACATCCTGTCCCCTCAGTTCATGTACGGCTCCAAGGCCTACGTGAAGCACCCCGCCGACATCCCCGACTACTTGAAGCTGTCCTTCCCCGAGGGCTTCAAGTGGGAGCGCGTGATGAACTTCGAGGACGGCGGCGTGGTGACCGTGACCCAGGACTCCTCCCTGCAGGACGGCGAGTTCATCTACAAGGTGAAGCTGCGCGGCACCAACTTCCCCTCCGACGGCCCCGTAATGCAGAAGAAGACCATGGGCTGGGAGGCCTCCTCCGAGCGGATGTACCCCGAGGACGGCGCCCTGAAGGGCGAGATCAAGCAGAGGCTGAAGCTGAAGGACGGCGGCCACTACGACGCTGAGGTCAAGACCACCTACAAGGCCAAGAAGCCCGTGCAGCTGCCCGGCGCCTACAACGTCAACATCAAGTTGGACATCACCTCCCACAACGAGGACTACACCATCGTGGAACAGTACGAACGCGCCGAGGGCCGCCACTCCACCGGCGGCATGGACGAGCTGTACAAGTAGGCGGCCGCTCTAGAACTAGTGGATCCACTTAACGTTACTGAAATCATCAAACAGCTTGACGAATCTGGATATAAGATCGTTGGTGTCGATGTCAGCTCCGGAGTTGAGACAAATGGTGTTCAGGATCTCGATAAGATACGTTCATTTGTCCAAGCAGCAAAGAGTGCCTTCTAGTGATTTAATAGCTCCATGTCAACAAGAATAAAACGCGTTTTCGGGTTTACCTCTTCCAGATACAGCTCATCTGCAATGCATTAATGCATTGACTGCAACCTAGTAACGCCTTNCAGGCTCCGGCGAAGAGAAGAATAGCTTAGCAGAGCTATTTTCATTTTCGGGAGACGAGATCAAGCAGATCAACGGTCGTCAAGAGACCTACGAGACTGAGGAATCCGCTCTTGGCTCCACGCGACTATATATTTGTCTCTAATTGTACTTTGACATGCTCCTCTTCTTTACTCTGATAGCTTGACTATGAAAATTCCGTCACCAGCNCCTGGGTTCGCAAAGATAATTGCATGTTTCTTCCTTGAACTCTCAAGCCTACAGGACACACATTCATCGTAGGTATAAACCTCGAAATCANTTCCTACTAAGATGGTATACAATAGTAACCATGCATGGTTGCCTAGTGAATGCTCCGTAACACCCAATACGCCGGCCGAAACTTTTTTACAACTCTCCTATGAGTCGTTTACCCAGAATGCACAGGTACACTTGTTTAGAGGTAATCCTTCTTTCTAGAAGTCCTCGTGTACTGTGTAAGCGCCCACTCCACATCTCCACTCGAAGTCGACGACAGCTAGCATCGATCTGACGATACTTTCTAGAGAATAGGAACTTCGGAATAGGAACTTCAAAGCGTTTCCGAAAACGAGCGCTTCCGAAAATGCAACGCGAGCTGCGCACATACAGCTCACTGTTCACGTCGCACCTATATCTGCGTGTTGCCTGTATATATATATACATGAGAAGAACGGCATAGTGCGTGTTTATGCTTAAATGCGTACTTATATGCGTCTATTTATGTAGGATGAAAGGTAGTCTAGTACCTCCTGTGATATTATCCCATTCCATGCGGGGTATCGTATGCTTCCTTCAGCACTACCCTTTAGCTGTTCTATATGCTGCCACTCCTCAATTGCTGCAGCTTTGGTTTAGGGTTAGGGCCCTCAGGGGCAGGGCATGCTCATGTAGAGCGCCTGCTCGCCGTCCGAGGCGGTGCCGTCGTACAGGGCGGTGTCCAGGCCGCAGAGGGTGAACCCCATCCGCCGGTACGCGTGGATCGCCGGTGCGTTGACGTTGGTGACCTCCAGCCAGAGGTGCCCGGCGCCCCGCTCGCGGGCGAACTCCGTCGCGAGCCCCATCAACGCGCGCCCGACCCCGTGCCCCCGGTGCTCCGGGGCGACCTCGATGTCCTCGACGGTCAGCCGGCGGTTCCAGCCGGAGTACGAGACGACCACGAAGCCCGCCAGGTCGCCGTCGTCCCCGTACGCGACGAACGTCCGGGAGTCCGGGTCGCCGTCCTCCCCGTCGTCCGATTCGTCGTCCGATTCGTCGTCGGGGAACACCTTGGTCAGGGGCGGGTCCACCGGCACCTCCCGCAGGGTGAAGCCGTCCCCGGTGGCGGTGACGCGGAAGACGGTGTCGGTGGTGAAGGACCCATCCAGTGCCTCGATGGCCTCGGCGTCCCCCGGGACACTGGTGCGGTACCGGTAAGCCGTGTCGTCGAGGGTGGCCATGAGATGCTTAGGTAGAATAGGTAAGTCAGATTGAATCTGAAATAAAGGGAGGAAGGGCGAACTTAAGAAGGTATGACCGGGTCGTCCACTTACCTTGCTTGACAAACGCACCAAGTTATCGTGCACCAAGCAGCAGATGATAATAATGTCCTCGTTCCTGTCTGCTAATAAGAGTCACACTTCGAGCGCCGCCGCTACTGCTACAAGTGGGGCTGATCTGACCAGTTGCCTAAATGAACCATCTAGTCAAACGACACAAATTTTGTGCTCACCGCCTGGACGACTAAACCAAAATAGGCATTCATTGTTGACCTCCACTAGCTCCAGCCAAGCCCAAAAAATGCTCCTTCAATATCAGTTACACTGGTGAGAATTCACCAGAACTGTCA
