# Supplementary material for: A protoplast generation and transformation method for soybean sudden death syndrome causal agents Fusarium virguliforme and F. brasiliense
Source: Fungal Biol Biotechnol. 2019 May 15;6:7. doi: 10.1186/s40694-019-0070-0 (PMC6518667; doi:10.1186/s40694-019-0070-0)
Supplement: Supplementary file 4 — Additional file 4. A supplementary file presenting a step-by-step protocol for protoplasting and transforming Fusarium virguliforme and Fusarium brasiliense. [file 40694_2019_70_MOESM4_ESM.pdf]

Title: A Protoplast Generation and Transformation Method for Soybean Sudden Death Syndrome Causal Agents *Fusarium virguliforme* and *F. brasiliense*

Purpose: Make fungal protoplasts (Part 1) that are competent for genetic transformation (Part 2)

Reference: Roth, M. G., and Chilvers, M. I. 2019. A Protoplast Generation and Transformation Method for Soybean Sudden Death Syndrome Causal Agents *Fusarium virguliforme* and *F. brasiliense*. Fung. Biol. and Biotech. XX:XXXX-XXXX.

Part 1. Protoplasting Materials

1. Potato Dextrose Broth (PDB)
   - 250 mL flasks filled with 50mL PDB (Neogen Corporation, Lansing, MI
2. Miracloth (Millipore-Sigma, Burlington, MA)
3. 30 µm nylon mesh filter (Millipore-Sigma)
4. Sterile molecular grade water, dH_2O
5. STC Buffer – 500 mL
   - 1.2 M D-Sorbitol
   - 10 mM $CaCl_2$
   - 10 mM Tris-HCl
   - pH to 7.5
6. Protoplasting solution buffer
   - 1.2 M KCl
7. Protoplasting solution – 30 mL
   - 30 mL of 1.2 M KCl
   - 750 mg Driselase from Basidiomycetes (Millipore-Sigma D8037)
   - 1.5 mg Chitinase from *Streptomyces griseus* (Millipore-Sigma Sigma C6137)
   - 150 mg lysing enzyme from *Trichoderma harzianum* (Millipore-Sigma L1412)

   Stir for 30 min and filter sterilize through a 0.45µm Millex-HA filter

Part 1. Protoplasting

1. Start a culture of *Fusarium* sp. on PDA. Let it grow until sporodochia are plentiful, and spores can be easily collected.
2. Flood the plate with 5 mL of sterile water (or 0.01% Triton X-100) and gently rub with a sterile spreader.
3. Re-collect as much of the solution as possible (typically ~ 4 mL), which now contains conidia. Use 100 µL to inoculate X number of 50 mL flasks of PDB.
4. Incubate flasks for 36-48 hours (maximum) at room temp, with shaking at 125 rpm.
5. Filter PDB cultures through sterile Miracloth and a Buchner funnel into a waste container.
6. Use a sterile spatula to scrape the mycelia from the Miracloth back into the 250 mL flask from which it came from (or a new sterile flask).
7. Add 30 mL of Protoplasting solution as quickly as possible to the mycelia, and incubate at 30°C for 3-5 hours, shaking at 75 rpm. Check them every 30 minutes to 1 hour. Incubate for up to 5 hours maximum.

8. Gravity filter the digested protoplasts through sterile 30 µm Nylon mesh filter and a Buchner funnel into a 50 mL conical tube.
9. Centrifuge tube at 3000 x *g* and 4°C for 5 minutes.
10. Carefully pour off supernatant. Gently resuspend protoplasts in 10 mL chilled STC buffer using wide orifice pipet tips. (Fully resuspending the protoplasts can be difficult, but still be gentle!)
11. Repeat the steps 9 and 10 once.
12. Repeat steps 9 and 10 again, but gently resuspend in 1 mL chilled STC buffer using a wide orifice pipet tip.
13. Quantify protoplasts using a hemocytometer. Make a 100X dilution (10 µL protoplasts in 990 µL STC). Take 10 µL of the dilution, inject it into a hemocytometer, and count cells under a microscope.
14. Dilute the 1 mL protoplast suspension with STC buffer to obtain 10^7 protoplasts per mL.
15. Use protoplasts immediately for transformation.
    Alternatively, aliquot 400 µL into as many tubes as possible, and add 30.1 µL DMSO to each (final concentration of 7% DMSO) and store directly in a -80°C freezer.

Part 2. Transformation Materials

1. Protoplasts
   - Either frozen or fresh
2. DNA fragments for transformation
3. Regeneration Medium (250mL)
   - 67.75 g Sucrose
   - 0.25 g Yeast Extract
   - 0.25 g N-Z Amine (Millipore-Sigma)
   - 1.86 g Agar
   - Autoclave (30 min, or else sucrose will caramelize)
4. 30% PEG solution (25 mL – Make fresh each time)
   - 7.5 g of PEG-8000 (Millipore-Sigma, Cat. No. P2139)
   - 10 mM Tris-HCl, pH 8.0
   - 50 mM $CaCl_2$
   - pH to 8.0
   - Stir for 30 minutes, and filter sterilize through 0.45 µm filter

Part 2. Transformation

1. At this point, you should have protoplasts (frozen or fresh) and a transformation construct. It is also recommended to make the Regeneration Media at this point and place it in a **<u>45°C</u>** water bath to begin cooling, but not solidifying.
2. Obtain protoplasts. If using frozen protoplasts, continue with step 3. If using fresh protoplasts, skip to step 6.
3. Obtain protoplasts from -80°C freezer and thaw on ice. Once thawed, centrifuge at 3000 x *g* for 4 minutes at 4°C.
4. Pipet off all supernatant and gently resuspend the pellet in 200 µL chilled STC using a wide orifice pipet tip.
5. Repeat the centrifugation and resuspension 2 more times.
6. Create a "transformation reaction" in this order:
   - 200µL of protoplasts in fresh STC buffer
   - 5 µg of linearized plasmid
     - should use less than 10 µL for this
   - 50 µL 30% PEG solution
   - about 250 µL total reaction volume (if >10 µL is needed when adding DNA construct, increase PEG volume so that PEG is 20% total reaction volume
   - Invert *gently* 2-3 times
7. Incubate the transformation reaction on ice for 1 hour. While waiting, add 2 mL of 30% PEG to a 15 mL conical tube, and 4 mL STC buffer to a different 15 mL conical tube. Keep both on ice.
8. *Gently* transfer the "transformation reaction" to the 2 mL of 30% PEG using a wide orifice pipet tip and incubate for 15 minutes on ice **<u>without mixing or inverting</u>**.
9. *Gently* pour the 4 mL of STC buffer into the transformation reaction and *gently* mix by inversion 2-3 times.
10. Immediately pour the protoplasts into 250 mL of regeneration media **<u>cooled to between 40-45°C</u>**.

11. Gently swirl the media until the protoplasts are evenly mixed in, then pour the media into as many Petri plates as possible (pour them thin, if possible).
12. Incubate the plates at room temperature for 24 hours.
13. While incubating, make 250 mL of regeneration media again, and cool to ~45°C. Add Hygromycin B to a final concentration of 100 µg / mL. Other antibiotics may be appropriate if using a different selectable marker gene.
14. Overlay the amended media over the transformation plates, but not so thick that the media will touch the lid.
15. Successful transformants may be seen growing through the amended media between 7-10 days.
16. Begin screening putative transformants by transferring to PDA amended with hygromycin.
17. Continue screening transformants via PCR and/or phenotyping.
